# Interplay between WO_6_ Octahedra Rigidity and Li Sub-Lattice Flexibility in Triclinic Li_2_W_2_O_7_: Raman, DFT, Hirshfeld Surface, and High-Pressure Studies

**DOI:** 10.1021/acs.inorgchem.6c01613

**Published:** 2026-06-15

**Authors:** José G. da Silva Filho, Gilberto D. Saraiva, Paulo T. C. Freire, João G. de Oliveira Neto, Daniel L. M. Vasconcelos, Romulo S. Silva, Lucas S. A. Olivier, Raí F. Jucá

**Affiliations:** † Faculty of Education, Sciences and Letters of the Sertão Central, State University of Ceará, 63902-098 Quixadá, Ceará, Brazil; ‡ Department of Physics, Federal University of Ceará, 60021-970 Fortaleza, Ceará, Brazil; § Center for Social Sciences, Health, and Technology, Federal University of Maranhão, Imperatriz, Maranhão 65900-410, Brazil

## Abstract

We report a combined experimental and theoretical investigation of the structural, electronic, elastic, and vibrational properties of triclinic Li_2_W_2_O_7_. The crystal structure was confirmed through Rietveld refinement, in good agreement with the reported triclinic model. Density functional theory (DFT) calculations within the DFT-GGA/PBE framework reproduce the experimental lattice parameters with deviations below 5%. Bader charge analysis reveals predominantly ionic Li–O interactions (Li ≈ +0.90*e*) combined with significant W–O covalency (W ≈ +2.98*e*), and electronic structure calculations show a wide O 2*p* → W 5*d* charge-transfer band gap characteristic of *d* ^0^ tungstates. Elastic constant calculations confirm mechanical stability with moderate anisotropy. Raman spectroscopy supported by DFT phonon calculations enables reliable mode assignment, revealing pronounced Li atomic motion in many WO_6_ vibrations. Hirshfeld surface analysis indicates that Li···O/O···Li contacts dominate the crystal packing (≈55.7%), with a 22.15% void fraction in the framework. High-pressure Raman measurements up to 9.3 GPa show predominantly positive pressure coefficients and clear spectral modifications between 6.3 and 7.5 GPa, providing evidence for a pressure-induced structural phase transition driven by octahedral tilting and symmetry reduction. The pressure response is governed by the interplay between rigid WO_6_ octahedra and a more compressible Li–O sublattice.

## Graphical abstract

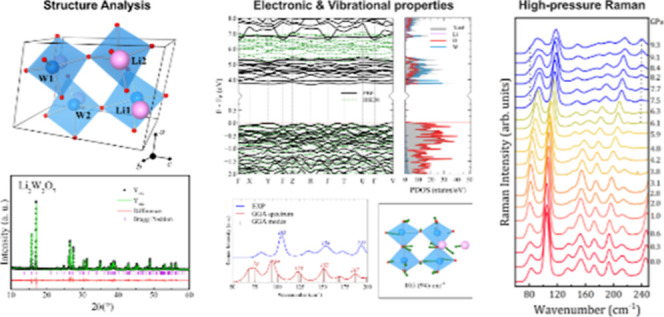

## 1 Introduction

The pyrochlore structure, with the general formula A_2_B_2_O_7_, represents a class of complex oxides capable of accommodating a wide variety of cations with distinct valences and ionic radii.^1,2^ In this framework, the site A may be occupied by mono-, di-, or trivalent cations, whereas site B is typically filled by transition metal cations, allowing for flexible charge balance and a broad diversity of chemical compositions.^3–6^ Structurally, pyrochlore compounds may be seen as ordered derivatives of the fluorite-type lattice or sal-gema, characterized by two distinct cationic sublattices and a partial ordering of anionic vacancies.^7,8^ This inherent structural versatility allows for significant chemical substitutions and local distortions, which exert a strong influence on the resulting physical and chemical properties. Consequently, A_2_B_2_O_7_ compounds exhibit remarkable multifunctionality, including magnetic ordering and frustration,^9,10^ ionic transport,^11,12^ dielectric behavior,^13–16^ and notable vibrational properties,^17,18^ rendering them attractive for both fundamental investigations and technological applications.

However, the structural complexity of these compounds renders Raman spectroscopy under extreme conditions (such as high pressure) an interesting tool for probing crystal lattice dynamics and structural stability in A_2_B_2_O_7_-type pyrochlore.^19–21^ In such systems, local symmetry breaking, BO_6_ octahedral distortions, and cation ordering enable a detailed analysis of pressure-induced wavenumber shifts, line width variations, and vibrational mode splitting. Therefore, the determination of pressure coefficients provides insight into bond compressibility, anharmonic effects, and crystal lattice stiffening mechanisms, thereby facilitating the identification of structural distortions and possible pressure-induced phase transitions.^22–25^ In these systems, phase transitions under compression are generally driven by the compressibility mismatch between the relatively rigid BO_6_ octahedral framework and the more deformable A–O sublattice, as well as by effects of the cation radius ratio, which affects defect formation energies and overall structural stability.^19–21^

According to reports in the literature, as pressure increases, bond shortening enhances lattice stiffness, while progressive cationic disorder, oxygen vacancy redistribution, and local symmetry breaking lead to the lifting of mode degeneracy, anomalous line width broadening, and changes in the behavior dω/d*P*.^26^ When the elastic stability of the ordered pyrochlore lattice is exceeded, reconstructive transformations toward defective fluorite or high-pressure cotunnite-type phases may occur, sometimes followed by pressure-induced amorphization resulting from accumulated stress and short-range disorder, as reported for rare earth pyrochlore^27^ and for Pb_2_Nb_2_O_7_.^28^ These characteristics render high-pressure Raman spectroscopy an interesting analysis for investigating compounds derived from distorted and low-symmetry pyrochlore, such as lithium tungstate Li_2_W_2_O_7_.^25^

As a member of the lithium–tungsten oxide family, Li_2_W_2_O_7_ is characterized by distorted WO_6_ octahedra and the presence of mobile Li^+^ cations, which give rise to complex lattice dynamics and pronounced phonon–phonon interactions.^29^ Structurally, it crystallizes in a triclinic lattice (space group *P*1̅ derived from an ideal pyrochlore structure, in which cation ordering, octahedral tilting, polymorphism, and intrinsic structural defects significantly influence on its physical properties.^29,30^ The high polarizability of the W–O bonds, combined with the mobility of lithium ions, renders this compound particularly attractive for applications in solid-state ionic conductors,^29^ electrochemical energy-storage systems,^31^ and dielectric devices.^32^

In this work, we present an experimental and theoretical investigation of Li_2_W_2_O_7_, combining DFT calculations for the assignment of vibrational modes with analyses of electronic band structure and density of states. Additionally, theoretical evaluations of elastic properties are conducted. Pressure-dependent Raman spectroscopy is further employed to probe the dynamics of the crystal lattice and elucidate the structural response of Li_2_W_2_O_7_ under extreme conditions, thereby providing a deeper understanding of its stability and phononic evolution.

## 2 Experimental Procedures

### 2.1 Synthesis of Pyrochlore-Type Li_2_W_2_O_7_

Pyrochlore-type Li_2_W_2_O_7_ was synthesized by the conventional solid-state reaction method using lithium carbonate (Li_2_CO_3_, Êxodo, 98%) and tungsten­(VI) oxide (WO_3_, Sigma-Aldrich, 99.99%) as starting materials without further purification. The reagents were weighed in stoichiometric proportions according to the reaction Li_2_CO_3_ + 2WO_3_ → Li_2_W_2_O_7_ + ↑CO_2_. Initially, the precursor powders were thoroughly mixed and ground in an agate mortar for approximately 30 min until a homogeneous mixture was obtained. The resulting powder was then placed in an alumina crucible and subjected to a preheating treatment at 550 °C for 6 h in a muffle furnace (7Lab) in order to promote the decomposition of Li_2_CO_3_ and the release of CO_2_. After this stage, the material was reground for an additional 30 min to further improve homogeneity and enhance solid-state diffusion. Finally, the powder was sintered at 660 °C for 6 h to ensure the complete formation of the Li_2_W_2_O_7_ phase.

### 2.2 Characterization Techniques

The structural characterization of pyrochlore Li_2_W_2_O_7_ was carried out by powder X-ray diffraction (PXRD) using Bragg–Brentano geometry. Diffraction data were collected with a PANalytical X’Pert Pro MPD diffractometer employing Co Kα radiation (λ = 1.7890 Å). The diffraction patterns were recorded over the 2θ range from 10° to 60°, with a step size of 0.013° and a counting time of 70 s per step. The crystal structure was refined by the Rietveld method using the GSAS-II software package,^33^ allowing the determination of crystal lattice parameters, atomic positions, and other structural parameters. Fourier transform infrared (FT-IR) measurements were performed on powder samples using a Bruker Vertex 70 spectrometer (Bruker, Rheinstetten, Germany), recorded in absorbance mode over the spectral range 60–4000 cm^–1^. Pressure-dependent Raman measurements of the Li_2_W_2_O_7_ pyrochlore were conducted using a membrane diamond anvil cell (mDAC), with mineral oil (Nujol) serving as the pressure-transmitting medium, covering pressures from ambient conditions (1 atm) up to 9.30 GPa. The spectra were collected using a Witec Alpha300 spectrometer equipped with a 532 nm excitation laser operating at approximately 20 mW. A 20× objective lens was used for signal acquisition, and the scattered light was dispersed by an 1800 grooves/mm grating, providing a spectral resolution of ±1 cm^–1^. Raman spectra were recorded within the spectral range from 60 to 1100 cm^–1^.

### 2.3 Computational Details

First-principles calculations based on DFT were carried out using the Quantum ESPRESSO package, version 7.3.1.^34,35^ The exchange–correlation energy was treated within the generalized gradient approximation (GGA) employing the Perdew–Burke–Ernzerhof (PBE) functional,^36^ while the electron–ion interactions were described using optimized norm-conserving Vanderbilt (ONCV) pseudopotentials from the SG15 library.^37^ The initial crystal structure of Li_2_W_2_O_7_ was obtained from the Crystallography Open Database (COD entry #2106564),^38^ corresponding to the experimental structure originally proposed by Okada et al.^25^ It is worth noting that Hämmer and Höppe^39^ recently reported a refined structural model in which the Li2 site exhibits partial positional disorder with a 73:27 occupancy ratio. However, since standard plane-wave DFT calculations cannot explicitly treat fractional occupancies, the present calculations were performed using the fully ordered model from the original determination. The electronic-wave functions were expanded in a plane-wave basis set with a kinetic energy cutoff of 120 Ry, and the Brillouin zone was sampled using a Γ-centered 4 × 3 × 3 Monkhorst–Pack *k*-point mesh.^40^ Full structural relaxation was performed using the BFGS algorithm within the variable-cell relaxation scheme, adopting convergence thresholds of 1.0 × 10^–8^ Ry for the SCF energy, 1.0 × 10^–4^ Ry/Bohr for the atomic forces, and 0.01 kbar for the stress components.

Following structural optimization, the electronic band structure was calculated along high-symmetry paths, while the density of states (DOS) and atom-projected DOS were computed using a denser *k*-point mesh with Gaussian smearing. Complementary fundamental band gap calculations were performed at the hybrid HSE06 level^41^ using maximally localized Wannier functions as implemented in the Wannier90 code.^42^ HSE06 incorporates a fraction of exact exchange and typically yields fundamental gaps closer to quasiparticle values than semilocal functionals; both PBE and HSE06 results are reported below as complementary estimates of the fundamental band gap rather than as a single best estimate. The effective atomic charges were determined using Bader’s quantum theory of atoms in molecules,^43^ providing insight into the relative ionic and covalent character of the chemical bonds. Elastic constants were calculated using the ElaStic code,^44^ from which the bulk, shear, and Young’s moduli were derived through the Voigt–Reuss–Hill averaging scheme.^45^

Phonon wavenumbers were computed within the framework of density functional perturbation theory (DFPT)^46^ as implemented in the PHonon package, and the Raman and infrared activities were determined from the derivatives of the polarizability and Born effective charge tensors.^47^ The phonon calculations were performed at the Brillouin zone center, which is the region directly relevant to the comparison with the Raman and FT-IR experimental data. To enable a quantitative comparison between the calculated and experimental vibrational wavenumbers, an optimal scale factor was determined following the procedure established by Scott and Radom.^48^ The scale factor λ was obtained by minimizing the root-mean-square deviation (RMSD) between the scaled calculated wavenumbers (λ·ω_calc_) and their experimentally assigned counterparts. The resulting value, λ = 1.043, yielded an RMSD of 7.64 cm^–1^ for the assigned mode pairs, representing a significant improvement relative to the unscaled wavenumbers. The optimal correspondence between calculated modes and experimental peaks was established using the Hungarian algorithm.^49^ Additional details on the scale factor optimization procedure, including correlation plots and residual analysis, are provided in the Supporting Information file (Figure S1).

The dominant atomic and internal-coordinate contributions to each normal mode were extracted from the DFPT eigenvectors using the vibrational mode automatic relevance determination (VMARD) scheme of the vibAnalysis program,^50^ a Bayesian sparse-regression decomposition mathematically equivalent in spirit to a Wilson-style potential energy distribution. The descriptions provided in Table S3 reflect this quantitative, mass-weighted output rather than a visual reading of the displacement vectors.

The three-dimensional (3D) Hirshfeld surface analysis was performed using the normalized contact distance parameter (*d*_norm_), which incorporates the distances from any point on the surface to the closest external nucleus (*d*_e_) and internal nucleus (*d*_i_), normalized by their corresponding van der Waals radii (*r*_vdW_). Complementary two-dimensional (2D) fingerprint plots were generated through the systematic mapping of *d*_e_ versus *d*_i_ parameters, providing a comprehensive coverage of all close contacts within the crystalline structure.^51^ Additionally, a void analysis within the primitive unit cell was conducted by examining electron-density isosurfaces at a threshold value of 0.01 au.^52^ All computational analyses were carried out using the CrystalExplorer software.^53^

## 3 Results and Discussion

### 3.1 Crystal Structure and Bader Charge Analysis

To validate the crystallographic model of Li_2_W_2_O_7_, room-temperaturePXRD data were analyzed by Rietveld refinement, as summarized in Figure 1a. The refinement was performed solely to confirm phase formation and consistency with the previously reported structural model, rather than to determine new atomic positions. The figure displays the experimental diffraction profile together with the calculated pattern from the refinement, exhibiting close agreement across the entire 2θ range.^6^ The difference curve remains of low magnitude, while the tick marks indicate the Bragg reflection positions indexed according to the refined phase. The quality of the refinement is supported by satisfactory agreement factors, with a weighted profile residual *R*_wp_ = 27.02% and a reduced chi-square value χ^2^ = 2.18, indicating a reliable correspondence between the observed and calculated patterns. Although the refinement shows good overall agreement, the relatively moderate *R*_wp_ value (∼27%) can be attributed to the complexity of the triclinic structure (*P*1̅), the large number of refined parameters, and microstructural effects such as microstrain (∼2074.5), which contribute to peak broadening and are not fully captured by the refinement model. Overall, the refinement confirms that Li_2_W_2_O_7_ crystallizes in the triclinic space group *P*1̅ (No. 2) with two formula per unit cell (*Z* = 2). The structural model, depicted in Figure 1b–d, reveals an anionic framework composed of two crystallographically distinct WO_6_ octahedra (W1 and W2) with different edge-sharing connectivity. These octahedra assemble into infinite [W_2_O_7_] double chains (ribbons), extending along the [100] direction and adopting a rock-salt-type arrangement. Lithium occupies two tetrahedrally coordinated sites (Li1 and Li2). A recent single-crystal analysis by Hämmer and Höppe^39^ revealed that Li2 exhibits partial disorder over two closely related configurations in a 73:27 ratio, which may contribute to the high ionic conductivity reported for this material.^54^ Since standard DFT calculations cannot explicitly treat fractional site occupancies, the present theoretical analysis was performed using the fully ordered structural model from the original determination by Okada et al.,^25^ as previously described, representing the majority Li_2_ configuration. Adopting this configuration captures the average WO_6_ framework geometry, W–O covalency, and Li–O coordination that govern the elastic, electronic and W-derived vibrational features reported below, but does not reproduce the configurational entropy of the Li_2_ sublattice nor the small (∼10 cm^–1^) inhomogeneous broadening that the 73:27 site mixing would introduce in Li-derived Raman modes; descriptions of modes with strong Li character should therefore be read as referring to the dominant ordered configuration. As illustrated in Figure 1d, the lithium environments can be described in terms of LiO_4_ tetrahedra. In the majority variant, these tetrahedra connect through corner-sharing into zigzag chains along the [100] direction, which further link to the surrounding [W_2_O_7_] ribbons to generate a 3D lattice. In contrast, the minority configuration may be rationalized by an alternative stacking arrangement of lithium–oxygen units along the same [100] direction.

**Figure 1:**
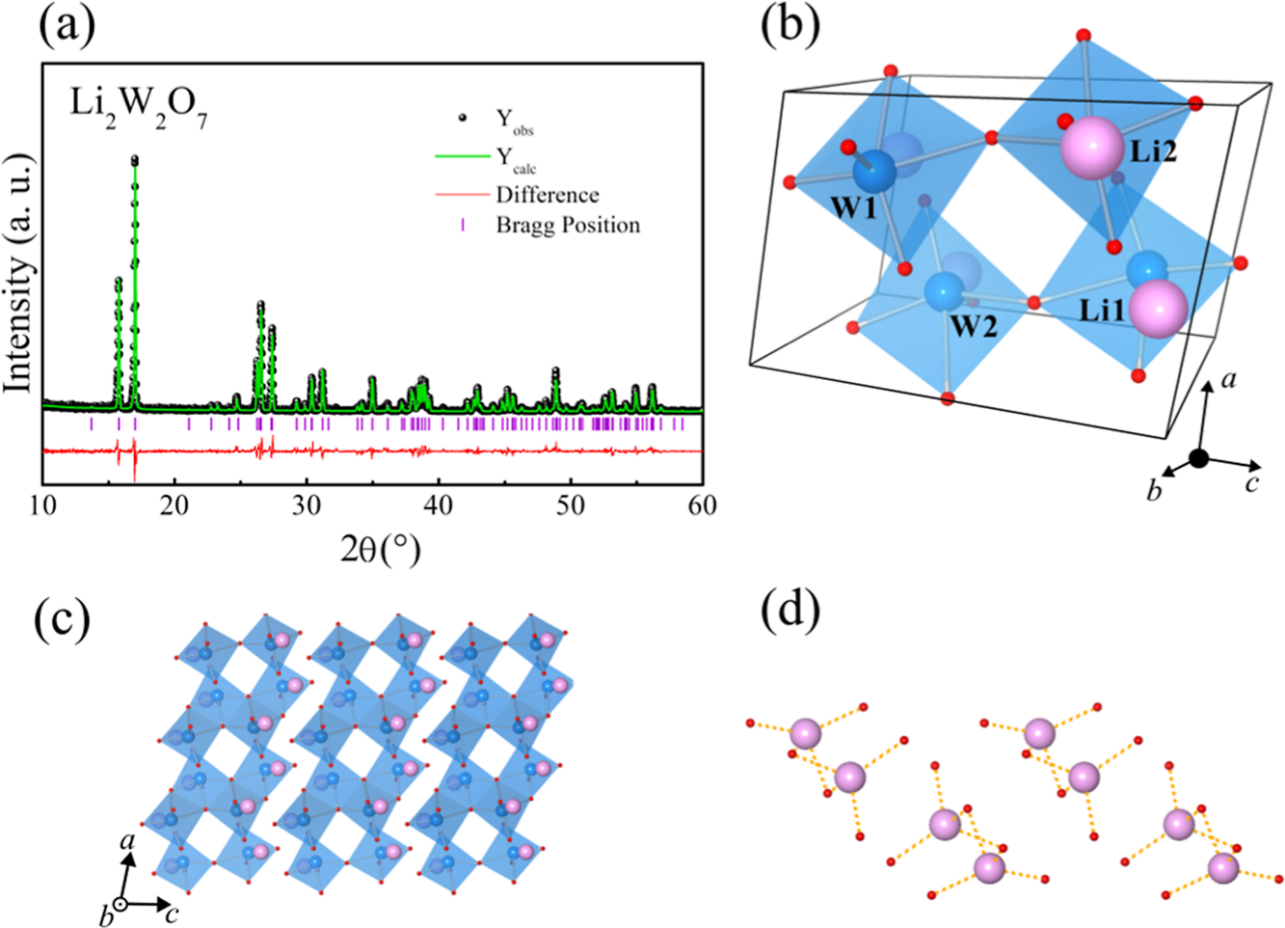
(a) Rietveld refinement of the PXRD pattern of Li_2_W_2_O_7_. Black symbols denote the observed intensities (*Y*_obs_), the green line corresponds to the calculated profile (*Y*_calc_), the red curve represents the difference (*Y*_obs_ – *Y*_calc_), and the magenta ticks indicate Bragg reflection positions. (b) Crystal structure of Li_2_W_2_O_7_, highlighting the WO_6_ octahedra (blue polyhedra) and the atomic sublattices (O in red; Li sites in pink). (c) Polyhedral representation emphasizing the connectivity of the tungstate framework, which can be described in terms of [W_2_O_7_] double chains running along the crystallographic [100] direction and linked into a three-dimensional network by the lithium sublattice. (d) Local lithium coordination motifs (LiO_4_ units; dashed lines indicate Li–O contacts), illustrating the tetrahedrally coordinated Li environments.

Table 1 summarizes the structural parameters optimized within the DFT-GGA/PBE framework. Following structural relaxation, an expanded lattice was obtained, with deviations of +1.67%, +0.64%, and +2.33% for the *a*, *b*, and *c* lattice parameters, respectively. The unit cell volume is overestimated by 4.56%, which is consistent with the well-documented underbinding behavior of GGA functionals, typically leading to volume expansions of about 3–4% in oxide materials.^54,55^ The calculated average W–O bond length of 1.978 Å shows excellent agreement with the experimental values (1.956 Å, deviation +1.10%), preserving the characteristic WO_6_ coordination geometry. The W–O distances span 1.739–2.317 Å, reflecting the highly distorted octahedra arising from their edge-sharing connectivity. Using the Balic-Zunic and Makovicky method,^56^ Hämmer and Höppe reported octahedral distortion parameters of Δ_oct_ = 0.2% for W1O_6_ (nearly regular) and Δ_oct_ = 9.7% for W2O_6_ (nonregular).^39^ The calculated O–W–O and W–O–W angles (103.50° and 113.73°) deviate by only +0.17% and +0.40% from experimental values, confirming the accurate reproduction of the local geometries.

**Table 1:** Experimental and DFT-GGA-Calculated Structural and Lattice Parameters, along with Effective Bader Charges of Li, W, and O Atoms in the Li_2_W_2_O_7_ System^a^

structural and lattice parameters	XRD	DFT-GGA	ΔGGA (%)
*a* (Å)	5.037	5.121	+1.67
*b* (Å)	7.050	7.095	+0.64
*c* (Å)	8.283	8.476	+2.33
α (°)	69.71	70.02	+0.44
β (°)	77.87	77.10	–0.99
γ (°)	85.40	84.27	–1.32
*V* (Å^3^)	269.72	282.02	+4.56
⟨W–O⟩ (Å)	1.956	1.978	+1.10
⟨O–W–O⟩ (°)	103.32	103.50	+0.17
⟨W–O–W⟩ (°)	113.27	113.73	+0.40
Total effective Bader charges (*e*)			
Li^+^	—	+0.90	
W^6+^	—	+2.98	
O^2–^	—	–1.10	

a The percentage deviation (Δ_GGA_) is provided to indicate the relative difference between the calculated and experimental values.

Bader charge analysis reveals distinct bonding regimes within the structure. Lithium atoms exhibit an effective charge of +0.90 *e* (90% of the formal +1 value), indicating predominantly ionic Li–O interactions consistent with other lithium oxides.^57^ In contrast, tungsten atoms show an effective charge of +2.98 *e* (50% of the formal +6 value), indicating substantial W–O covalency. Oxygen atoms accumulate −1.10 *e* (55% of the formal −2 value). These results parallel the Bader analysis of isostructural compounds: Jucá et al. reported effective charges of +3.11 *e* for Mo^6+^ (52%) and −1.27 *e* for O^2-^ in β-Bi_2_Mo_2_O_9_,^58^ while Wang et al. identified a comparable M–O covalency in scheelite-type tungstates, where the valence band is predominantly composed of O 2*p* states and the conduction band is mainly derived from W 5*d* orbitals.^59^ This dual bonding character, ionic Li–O combined with covalent W–O bonding, is characteristic of alkali metal tungstates and underlies both the wide band gap^60^ and the ionic conductivity properties relevant for solid-state battery applications.^29,31^

To go beyond the effective charges and quantify how much of the W–O charge transfer is genuinely ionic versus polar-covalent, we performed a Bader integration of the charge density difference Δρ = ρ_crystal_ – Σρ_atom_ over the same atomic basins, obtaining a mean local change of only −0.78 *e* per W atom compared with the total Bader transfer of −2.98 *e*. The ratio |Δρ|/|Δ*q*| ≈ 0.26 indicates that ≈74% of the W charge that is removed relative to the isolated neutral atom remains distributed in the W–O interatomic region rather than entering the O basins, a direct quantitative signature of W–O covalency, while the corresponding ratio for Li (≈0.04) is consistent with predominantly ionic Li–O bonding. The electron localization function (ELF) (Figure S3b) confirms this picture, showing high ELF values (≈0.85) along the W–O bonds and on the oxygen lone pairs in contrast to low ELF (≈0.4) between Li and O.

### 3.2 Elastic Properties

The elastic constants of Li_2_W_2_O_7_ were determined using the ElaStic code,^44^ which evaluates the second-order elastic stiffness tensor C_ij_ from stress–strain relationships. For the triclinic system (space group P1̅) the elastic tensor contains 21 independent components. The effective polycrystalline elastic moduli were subsequently derived using the Voigt–Reuss–Hill averaging scheme^64^ which provides the upper (Voigt) and lower (Reuss) bounds of the elastic moduli; the Hill averages yield the best estimates for the polycrystalline bulk and shear moduli (the explicit Voigt–Reuss–Hill expressions are given in the Supporting Information).

The mechanical stability was verified by confirming that all eigenvalues of the elastic constant matrix are positive.^61^ The calculated elastic parameters are summarized in Table 2. All six eigenvalues are positive, confirming that the triclinic Li_2_W_2_O_7_ structure is mechanically stable under ambient conditions.

**Table 2:** Calculated Elastic Parameters of Li_2_W_2_O_7_: Eigen Values of the Elastic Constant Matrix (λ_i_), Voigt (V), Reuss (R), and Hill Averages for Bulk (B) and Shear (G) Moduli, Young’s Modulus (E), Poisson’s Ratio (ν), and Universal Anisotropy Index (A^U^)­^a^

λ_1_	λ_2_	λ_3_	λ_4_	λ_5_	λ_6_	*B*_V_	*B*_R_	*B*	*G*_V_	*G*_R_	*G*	*E*	ν	*A* ^U^
179.7	86.2	48.7	41.1	28.5	14.7	59.2	57.4	58.3	31.1	25.5	28.3	73.1	0.29	1.12

a All moduli are in GPa.

The calculated bulk modulus, *B* = 58.3 GPa, represents the material’s resistance to hydrostatic compression, a key parameter for understanding its structural response under high-pressure conditions. This value is comparable to those reported for related tungstate materials like scheelite-type BaWO_4_ exhibits *B*_0_ ∼ 52 GPa,^62,63^ while CaWO_4_ shows *B*_0_ ∼ 75 GPa^64,65^ and PbWO_4_ has *B*_0_ ∼ 63 GPa.^66^ The intermediate bulk modulus of Li_2_W_2_O_7_ reflects its structural characteristics, combining relatively rigid WO_6_ octahedral units with more compressible Li–O coordination environments. The shear modulus, *G* = 28.3 GPa, and Young’s modulus, *E* = 73.1 GPa, quantify the resistance to shear deformation and uniaxial stress, respectively. The Poisson’s ratio, ν = 0.29, indicates a moderate lateral expansion upon axial compression, a behavior typically observed in ionic–covalent oxide materials. The moderate magnitude of the bulk modulus further suggests that Li_2_W_2_O_7_ should exhibit measurable structural changes under externally applied pressures in the GPa range, rendering it a suitable candidate for high-pressure Raman spectroscopy investigations aimed at exploring pressure-induced phase transitions.

The elastic anisotropy was further quantified using the universal anisotropy index *A* ^U^ (see Supporting Information for the explicit expression; the full elastic stiffness and compliance tensors are reported in Tables S1 and S2). The calculated value *A* ^U^ = 1.12 indicates a moderate anisotropy, since a value of zero corresponds to elastic isotropy. This anisotropic behavior arises from the low symmetry of the triclinic structure and from the directional character of the covalent W–O bonds combined with the more flexible ionic Li–O coordination. Consequently, the material is expected to exhibit nonuniform compression under hydrostatic pressure, with different crystallographic directions exhibiting distinct compressibility. Such type of anisotropic response is commonly observed in low-symmetry tungstates and may influence the pathway of pressure-induced structural transformations.^59^

### 3.3 Hirshfeld Surface and Crystal Voids Analysis

To gain deeper insight into the structural framework and the intermolecular interactions governing the crystal packing of Li_2_W_2_O_7_, a comprehensive 3D Hirshfeld surface analysis combined with 2D fingerprint plots was conducted. This approach provides a quantitative visualization of close intermolecular contacts within the crystal structure and their relative contributions to the overall crystal packing stability. The full Hirshfeld surface mapped over *d*_norm_ (Figure 2) reveals distinct regions color-coded according to contact distances: red areas indicate contacts shorter than the sum of r_vdW_ (close contacts), white regions represent contacts approximately equal to r_vdW_ separations, and blue areas correspond to contacts longer than r_vdW_ (distant contacts).^67^ The accompanying 2D fingerprint plot displays the distribution of all intermolecular contacts as a function of *d*_e_ versus *d*_i_, providing a characteristic signature of the crystal packing arrangement.^68^

**Figure 2:**
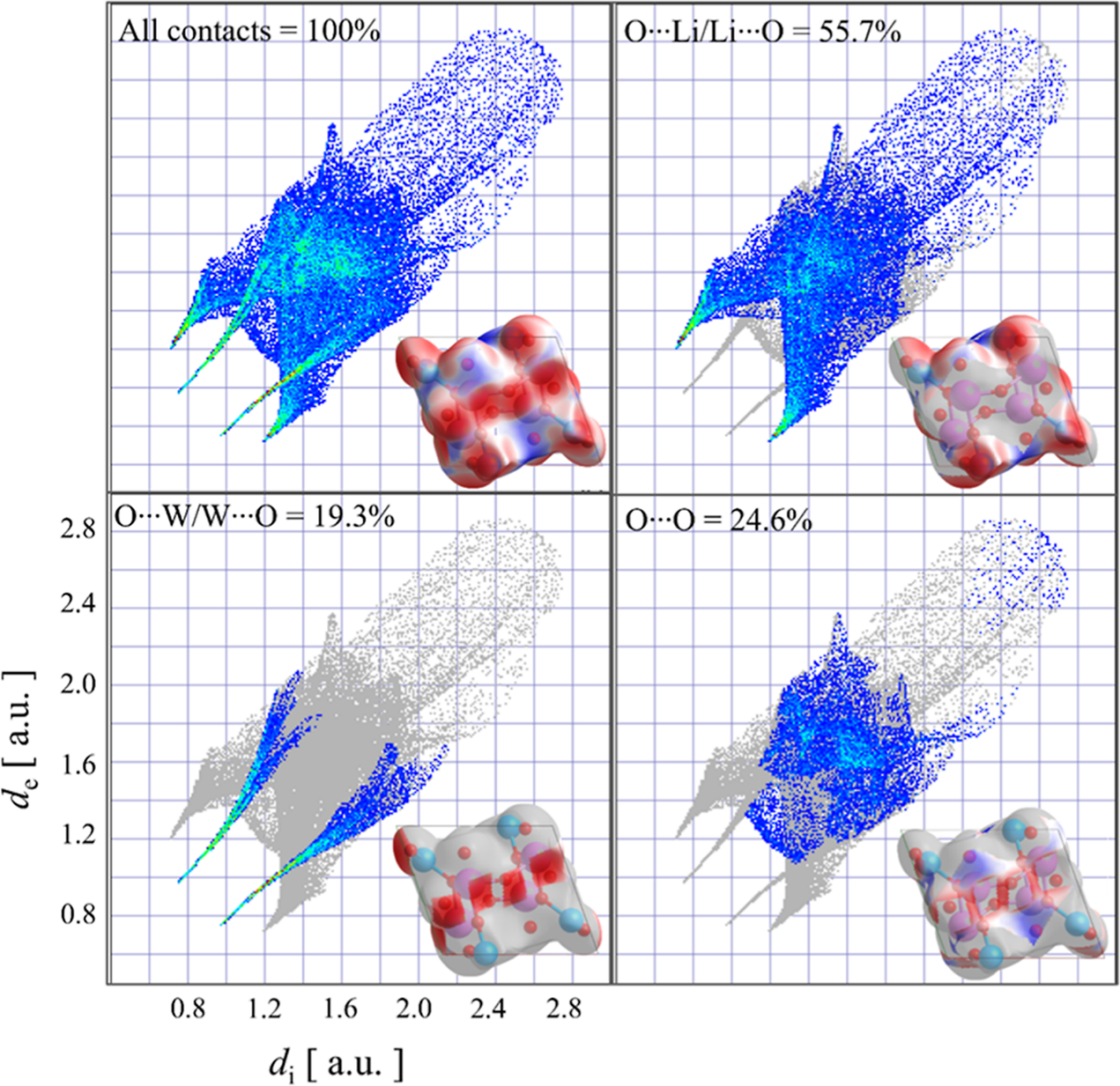
2D fingerprint plots (total and specific) and 3D Hirshfeld surfaces of the Li_2_W_2_O_7_ framework.

As illustrated, the predominant interaction within the Li_2_W_2_O_7_ structure corresponds to O···Li/Li···O contact, accounting for 55.7% of the total Hirshfeld surface area. The surface exhibits prominent red regions surrounding both oxygen and lithium atoms, signifying close ionic contacts that are significantly shorter than the sum of *r*_vdW_. These interactions are primarily electrostatic in nature, reflecting the strong ionic bonding between Li^+^ cations and O^2–^ anions that constitute the fundamental building blocks of the pyrochlore framework. The substantial contribution and close contact distances underscore the key role of Li–O coordination in stabilizing the 3D lattice. The geometry of these Li···O/O···Li contacts is consistent with the structural prerequisites for Li^+^ migration discussed for related lithium tungstates,^69,70^ but since no transport experiment is reported here this connection is offered as a structural rationale rather than as a demonstrated functional property.

The second most significant contribution arises from O···W/W···O contacts (19.3%), which manifest exclusively as red regions on the Hirshfeld surface and are accompanied by characteristic sharp spikes at low values of both *d*_e_ and *d*_i_ in the fingerprint plot, together with scattered red points. These features are indicative of very short and directional metal–oxygen coordination bonds within the WO_6_ octahedral units that constitute the pyrochlore framework. The sharp spikes at low *d*_e_ + *d*_i_ values reflect the strong covalent-ionic character of W–O bonds, which are substantially shorter and stronger than typical van der Waals interactions. These W–O interactions are fundamental to the structural rigidity of the pyrochlore lattice, providing the framework necessary to preserve structural integrity at elevated temperatures and under demanding operational conditions in catalytic or photocatalytic applications.^39,71^

The remaining contributions to the Hirshfeld surface arise from O···O contacts (24.6%), reflecting moderate-range oxygen–oxygen interactions that contribute to the overall packing efficiency, and from Li···Li contacts (0.4%), confirming that the Li^+^ cations maintain sufficient separation to minimize cation–cation repulsions. A detailed discussion of these secondary interactions is provided in the Supporting Information.

Collectively, the Hirshfeld surface analysis reveals that the Li_2_W_2_O_7_ pyrochlore structure is stabilized primarily through strong ionic Li···O/O···Li interactions (55.7%) and robust W···O/O···W coordination bonds (19.3%), complemented by secondary contributions from O···O contacts (24.6%). This distribution of intermolecular interactions reflects the highly ionic nature of the material and the 3D connectivity that characterizes the pyrochlore architecture.

To investigate the void space distribution within the Li_2_W_2_O_7_ pyrochlore structure, a comprehensive analysis of the crystalline packing was conducted using electron-density isosurfaces set at 0.01 au Figure 3 presents multiple orientations of the void distribution within the primitive unit cell, revealing the spatial arrangement and structural characteristics of the unoccupied regions.

**Figure 3:**
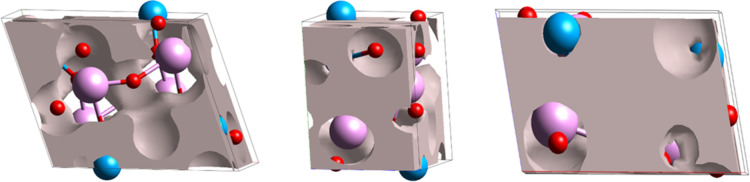
Crystal void analysis of Li_2_W_2_O_7_ showing voids distribution in the primitive unit cell from multiple orientations.

The quantitative analysis indicates that the primitive unit cell possesses a total volume of 267.23 A^3^, with void regions accounting for 59.19 A^3^, corresponding to 22.15% of the total cell volume. The calculated isosurface area amounts to 142.91 A^2^. The void geometry was further characterized by globularity and asphericity parameters of 0.514 and 0.176, respectively. A globularity value of ∼0.5 suggests moderately spherical voids, while the relatively low asphericity indicates limited deviation from ideal spherical geometry.^72^ The 22.15% void fraction and the moderate sphericity of the cavities are consistent with an open framework that could in principle accommodate Li^+^ migration pathways; whether these voids form percolating channels suitable for fast-ion transport cannot be established from static-structure geometry alone and would require dedicated activation-barrier or impedance-spectroscopy studies.^73^

### 3.4 Vibrational Study and Group Theory

Li_2_W_2_O_7_ crystallizes in the triclinic space group *P*1̅ (No. 2) with *Z* = 2 and all atoms at the general Wyckoff position 2i. The factor group is isomorphic to the point group *C*_i_ (or S_2_), with the inversion center as the sole symmetry element.^74,75^ Applying the correlation method to each 2i pair, the optical representation decomposes as Γ_optic_ = 33*A*_g_ + 30*A*_u_, with the 33 A_g_ modes Raman-active and the 30 A_u_ modes IR-active, in accordance with the mutual exclusion rule.^76^

Figure 4a,b shows a comparison between the experimental Raman spectra (blue curve) and those calculated within the GGA approximation (red curve) for pyrochlore Li_2_W_2_O_7_, separated into low- and high wavenumbers regions. A good agreement is observed between the positions of the experimental vibrational modes and those predicted theoretically, with minor discrepancies attributable to approximations inherent to the exchange–correlation functional and the experimental conditions.

**Figure 4:**
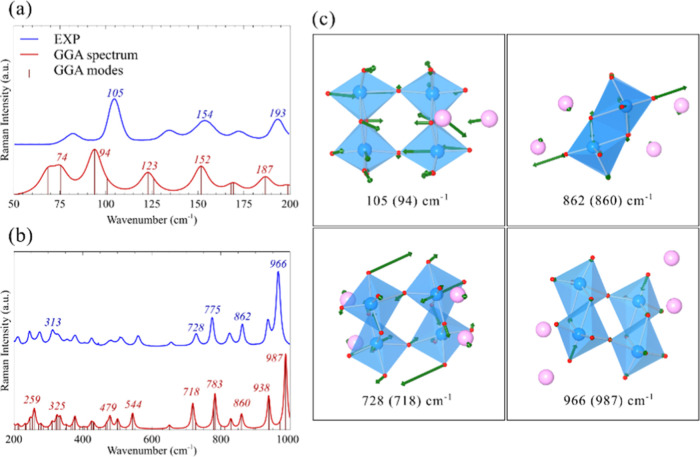
Experimental (blue) and calculated (red) Raman spectra of Li_2_W_2_O_7_ in the ranges of (a) 50–200 cm^–1^ and (b) 200–1000 cm^–1^. Calculated vibrational wavenumbers were uniformly scaled by a factor λ = 1.043 to facilitate comparison with experimental data. Vertical bars indicate the positions of the calculated Raman-active modes. (c) Schematic atomic-displacement patterns (vibrational eigenmodes) for selected Raman-active modes at 105, 728, 862, and 966 cm^–1^. For each mode, the value in parentheses corresponds to the scaled GGA wavenumber.

Furthermore, Figure 4c illustrates the atomic-displacement patterns of four representative Raman-active modes, all of A_g_ symmetry, that span the full spectral range: a low-frequency translational mode at 105 cm^–1^ (WO_6_ unit translation coupled to Li motion), a W–O–W bridge stretching mode at 728 cm^–1^ with moderate W and Li displacement, and two high-frequency W–O stretching modes at 862 and 966 cm^–1^. The complete list of all 33 Raman-active (A_g_) and 30 IR-active (A_u_) zone-center modes, with their irreducible-representation labels, calculated and experimental wavenumbers, and dominant atomic motions, is presented in Table S3. The corresponding comparison between experimental and calculated infrared (IR) spectra is presented in Figure S2 of the Supporting Information, confirming the consistency of the DFPT phonon calculations across both Raman- and IR-active modes.

### 3.5 Electronic Properties

The electronic band structure and DOS of Li_2_W_2_O_7_ were computed at both the PBE and HSE06 levels of theory. Figure 5a displays the first Brillouin zone of the triclinic lattice, highlighting the labeled high-symmetry points. The band structure was calculated along the path G–X–Y–G–Z–R–G–T–U–G–V using both PBE and HSE06 functionals.^41,42^ Figure 5b,c present the resulting band structures, together with the total and atom-projected density of states (PDOS), as well as the orbital-resolved PDOS for each constituent element. Both levels of theory predict an indirect band gap, with the valence band maximum (VBM) located at the V point and the conduction band minimum (CBM) at the R point. The calculated fundamental band gap along the band-structure path is 3.67 eV at the PBE level and 5.13 eV at the HSE06 level. The PBE → HSE06 increase of approximately 40% reflects the partial inclusion of exact exchange in HSE06; both values represent vertical band-to-band (Kohn–Sham/quasiparticle-like) transitions and should be regarded as complementary estimates of the fundamental gap rather than as a single best value.

**Figure 5:**
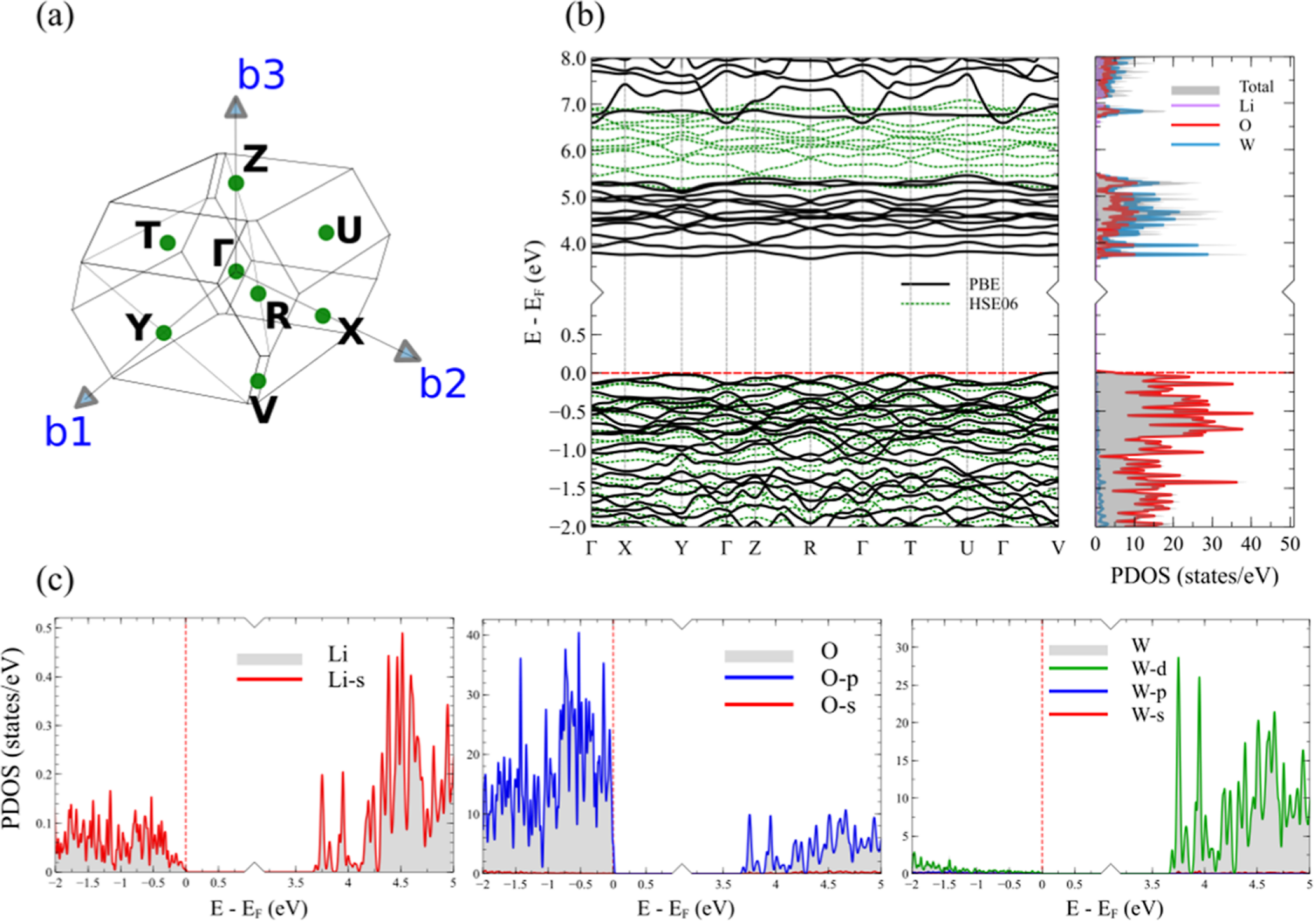
(a) First Brillouin zone of Li_2_W_2_O_7_ (primitive cell), indicating the reciprocal lattice vectors b1–b3 and the high-symmetry points employed to define the band-structure path Γ–X–Y–Γ–Z–R–Γ–T–U–Γ–V. (b) Electronic band structure calculated using the PBE (solid black) and HSE06 (dashed green) functionals, together with the total and element-resolved DOS. (c) Orbital-projected density of states (PDOS) for Li, O, and W, decomposed into Li-*s*, O-*s*, O-*p*, W-*s*, W-*p*, W-*d* contributions. Energies are referenced to the Fermi level (*E* – *E*_F_ = 0), indicated by the red dashed line.

Both the highest valence band and the lowest conduction band are remarkably flat, with bandwidths of only ∼0.15 eV each, which leads to a small difference between the indirect and the lowest direct gap (∼0.09 eV). The indirect nature of the fundamental gap implies that the lowest-energy electronic transitions require phonon assistance, which affects the optical absorption onset of the material. Experimentally, Tabero and Frąckowiak^77^ reported an optical band gap of 4.03 eV from UV–Vis–NIR diffuse reflectance spectroscopy (DRS) measurements on Li_2_W_2_O_7_ powders, extracted via Tauc/Kubelka–Munk analysis under the assumption of an indirect transition. We emphasize that the experimental optical gap and the fundamental band gaps reported above are not formally equivalent quantities and should not be expected to coincide. The fundamental gap is the energy of a vertical electronic transition between the highest occupied and lowest unoccupied single-particle states, whereas the optical gap is the onset of optical absorption and is systematically reduced by (i) the binding energy of the lowest excitonic state, (ii) electron–phonon renormalization of the band edges, and (iii) the choice of fitting model in the Tauc plot for indirect-gap systems.^59,60,78^ For wide-gap *d* ^0^ tungstates, exciton binding energies of several hundred meV and electron–phonon renormalizations of comparable magnitude have been reported in the literature, which together can account for shifts of the order of 1 eV between the fundamental and optical gaps. We therefore refrain from interpreting either the PBE underestimate or the HSE06 overestimate of the optical value as a direct measure of functional accuracy. A rigorous quantitative comparison between calculated and measured gaps would require a many-body GW + Bethe–Salpeter calculation, which lies beyond the scope of the present work but represents a natural extension.

The orbital-PDOS (Figure 5b,c) shows that the upper valence band, spanning approximately 6 eV below the Fermi level, is dominated by O 2*p* states with a secondary W 5*d* contribution from W–O covalent hybridization, while the topmost states are essentially nonbonding O 2*p* lone pairs. The lower conduction band is composed of empty W 5*d* states with substantial O 2*p* admixture, reflecting the antibonding counterpart of the W–O interaction. The W 5*d* partial DOS displays a multipeaked profile spanning approximately 5 eV above the CBM, arising from the markedly different distortion of the two crystallographically distinct W environments (W1 and W2),^25,39^ which lifts the degeneracy of the 5*d* manifold beyond the t_2__g_/e_g_ splitting of an ideal octahedral field. The persistent W 5*d* contribution within the valence band confirms W 5*d*–O 2*p* covalent hybridization, consistent with the Bader effective charges (W + 2.98 *e*, Li + 0.90 *e*; Section 3.1). Overall, the electronic structure is characterized by an O 2*p* → W 5*d* charge-transfer gap, the hallmark of *d* ^0^ transition-metal oxides.^79,80^

The wide indirect gap of Li_2_W_2_O_7_ is consistent with the general picture for *d* ^0^ tungstates: scheelite-type CaWO_4_ (4.94 eV experimental^79^), BaWO_4_ (5.26 eV), and spinel-type Na_2_WO_4_ (4.4 eV calculated within the FP-LAPW framework^81^). These contrast with tungstates containing partially filled *d* shells, where transition-metal d states reduce the gap and contribute to the band edges: NiWO_4_ (∼3.0 eV indirect, with Ni 3*d* at the VBM, gap decreases under compression^82^), FeWO_4_ (∼2.0 eV, Fe 3*d* dominating the VBM^83^), and γ-Fe_2_WO_6_ (1.55 eV at the PBE level^84^).

### 3.6 Pressure-Dependent Raman Study

The pressure evolution of the Raman-active modes reveals a complex behavior characterized by mode hardening, slight softening at low wavenumbers, and the emergence, disappearance, and splitting of modes, as well as variations in the intensities of the Raman bands and the appearance of additional modes at higher pressures (*P* ≤ 9.3 GPa). The Raman spectra are represented by a color gradient from red to yellow, indicating the gradual change in Raman intensity, followed by blue, where the phase transition takes place. The appearance of modes is indicated by dotted lines in the Raman spectra and by semifilled stars in the wavenumber versus pressure plots on the right-hand side, where the red and blue transparent rectangles represent different phases. Figure 6a,b show the low wavenumber modes located at 82.4 and 153.1 cm^–1^ (at ambient pressure) initially soften slightly up to ∼1–1.3 GPa at (82.4 → 80.0 cm^–1^) and (153.1 → 152.7 cm^–1^), respectively, suggesting enhanced lattice instability or increasing anharmonicity in external (translational) modes. After ∼2–3 GPa, these modes reverse behavior and harden continuously, reaching 92.2 and 177.0 cm^–1^ at 9.3 GPa. Such nonmonotonic evolution is often indicative of subtle structural rearrangements or changes in the compressibility of specific lattice directions. At low pressures (0–2 GPa), most phonon modes exhibit a positive pressure coefficient (dω/d*P* > 0), indicating lattice stiffening under compression. Representative examples include the modes near 135 cm^–1^ (134.1 → 136.6 cm^–1^), 193 cm^–1^ (193.1 → 195.9 cm^–1^), 275 cm^–1^ (275.1 → 278.7 cm^–1^), 377 cm^–1^ (377.5 → 389.4 cm^–1^), and 560 cm^–1^ (560.5 → 569.9 cm^–1^). The intermediate wavenumbers modes (∼280–570 cm^–1^), typically associated with internal bending vibrations of polyhedral units, exhibit larger absolute shifts, consistent with progressive bond shortening and increased force constants under compression. Between 2 and 6 GPa, the majority of modes continue to harden in a quasi-linearly manner, with moderate pressure coefficients. For instance, the mode near 404 cm^–1^ increases from 404.1 cm^–1^ (0 GPa) to ∼405–410 cm^–1^ (6 GPa), and to ∼422 cm^–1^ at 9.3 GPa. Similarly, the highest-wavenumbers mode evolves from ∼560.5 cm^–1^ at ambient pressure to ∼589.7 cm^–1^ at 9.3 GPa, indicating a pronounced stiffening of the strongest bonds in the structure.

**Figure 6:**
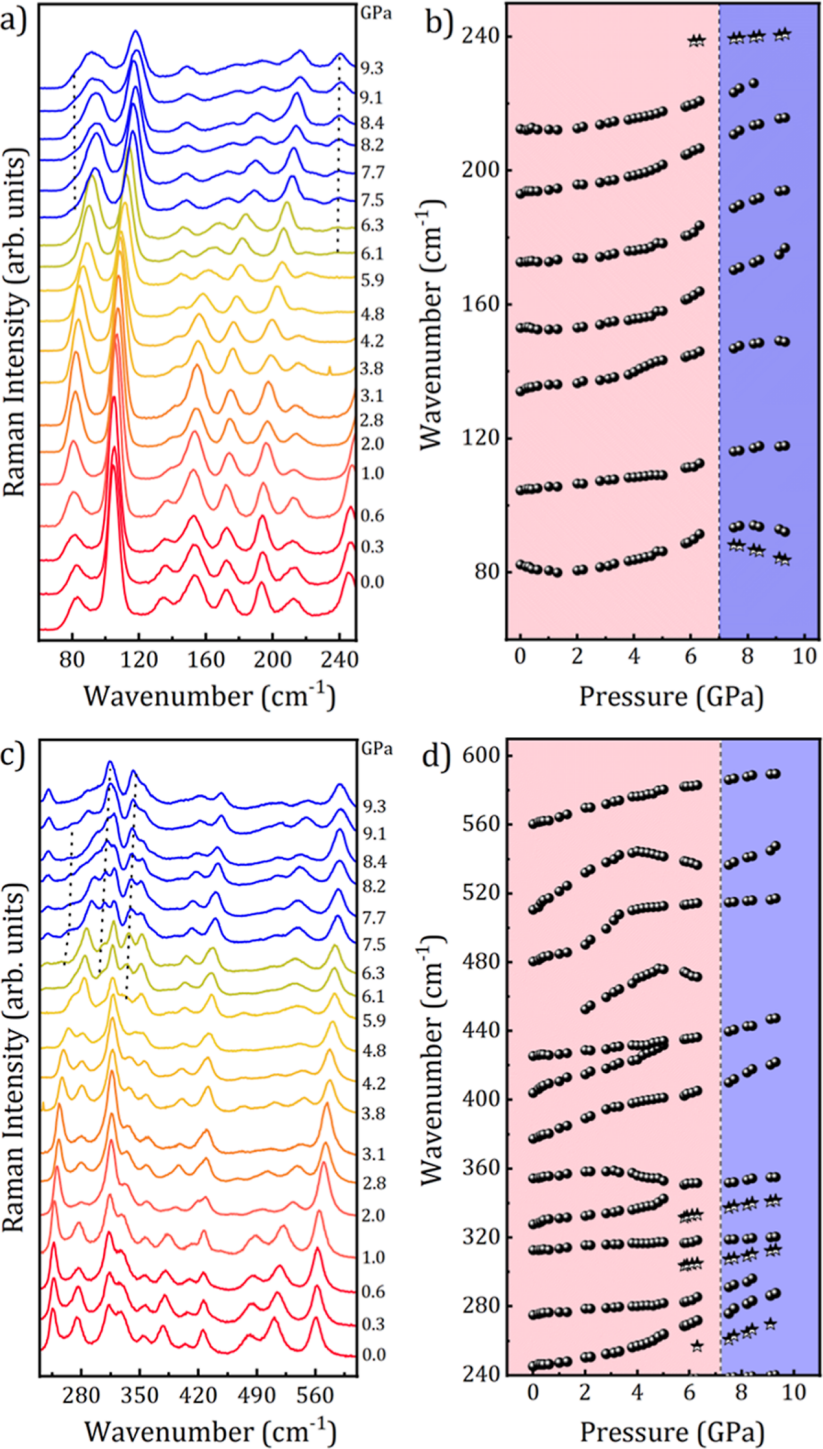
Pressure-dependent Raman spectra of Li_2_W_2_O_7_ in the following spectral regions: (a) 50–260 cm^–1^ and (c) 230–610 cm^–1^. Wavenumber versus pressure plots of the Raman modes of Li_2_W_2_O_7_ crystals in the corresponding spectral regions are shown in (b) 60–250 cm^–1^ and (d) 240–610 cm^–1^.

Above the pressure range of 2.0–6.3 GPa, noticeable spectral modifications become evident. New modes appear at 88.3, 238.7, 257.2, 303.8, 331.8, and 435.3 cm^–1^. Additionally, several wavenumber shifts become nonlinear, particularly for modes around 320–520 cm^–1^. All these features strongly suggest the onset of a pressure-induced structural phase transition, which is most likely associated with a symmetry change from the initial centrosymmetric phase *P*1̅ to a lower-symmetry or symmetry-modified structure, possibly involving the loss of inversion symmetry (*P*1) and/or an increase in the number of crystallographically independent units (WO_6_) in the unit cell. It is well-known that a phase transition from *P*1̅ to *P*1 relaxes the Raman selection rules, which may allow the appearance of additional Raman modes in the spectra. The limited emergence of new Raman modes implies that the inversion symmetry breaking involves only subtle structural distortions. At room temperature, Li_2_W_2_O_7_ crystallizes in the triclinic space group *P*1̅ (*C*_i_^1^), with all atoms at the general Wyckoff position “2i”. However, a system can evolve under pressure while remaining in the same space group (e.g., *P*1̅ → *P*1̅.). The evolution may involve a redistribution of atoms between general and special Wyckoff positions, leading to an increase in crystallographically independent sites while preserving the *P*1̅ (C_i_^1^) symmetry. Such isostructural changes can lead to modifications in the Raman spectra, including the appearance of additional modes, without requiring a change in the space group symmetry. In this study, upon increasing pressure, the system may evolve within the triclinic centrosymmetric space group *P*1̅ (C_i_^1^). In this phase, atoms can occupy either the special positions (*a*, *b*), with site symmetry *C*_i_, or the general position 2i, with site symmetry C_1_. This leads to configurations in which atoms are distributed between general and inversion-center sites. More generally, the *P*1̅ space group allows structural arrangements combining atoms at inversion centers and at general positions, resulting in different numbers of crystallographically independent sites. In summary, the *P*1̅ space group hosts atoms, which may occupy either the special positions “*a*” or “*b*” with site symmetry C_i_, or the general position 2i with site symmetry C_1_, leading to configurations of the type (a + b) C_i_(1) or [2i C_1_(2)] + (a) C_i_(1). More generally, the *P*1̅ space group allows Wyckoff arrangements of the form [2i *C*_1_(2)] + (*h* + *g* + *f* + *e* + *d* + *c* + *b* + *a*) *C*_i_(1). Additional experimental and theoretical efforts are needed to fully elucidate the character of this transition and its effects on lattice dynamics.

It is worthwhile to say that a phase transition is identified based on well-defined and collective changes in the lattice dynamics, particularly in the low-wavenumbers Raman region (below 200 cm^–1^), which is directly associated with external (lattice) modes involving translational and rotational motions of structural units. However, we attribute these changes above 200 cm^–1^ as a conformational change, due to the rearrangements or local structural distortions rather than a full phase transition, since they do not involve a clear reorganization of the lattice modes. In fact, conformational changes have been reported to precede the phase transition in certain tungstate and molybdate compounds^85,86,87,88,89,90^ , leading to a structural transformation as pressure increases. In the present case, changes in the lattice modes occur within the 6.3–7.5 GPa range, where the phase transition takes place. The emergence of additional Raman bands implies an increase in the number of Raman-active modes, consistent with a modification of the space group of the crystal symmetry. At the highest pressures (7.5–9.3 GPa), several modes exhibit altered slopes and partial softening (e.g., the ∼93 cm^–1^ mode slightly decreases from 94.2 to 92.2 cm^–1^), further supporting the establishment of a distinct structural regime. Meanwhile, the high-wavenumbers modes (>500 cm^–1^, in Figure 7a,b) continue to harden, although with reduced pressure coefficients in certain cases, suggesting changes in compressibility or in the local bonding environment within the high-pressure phase.

**Figure 7:**
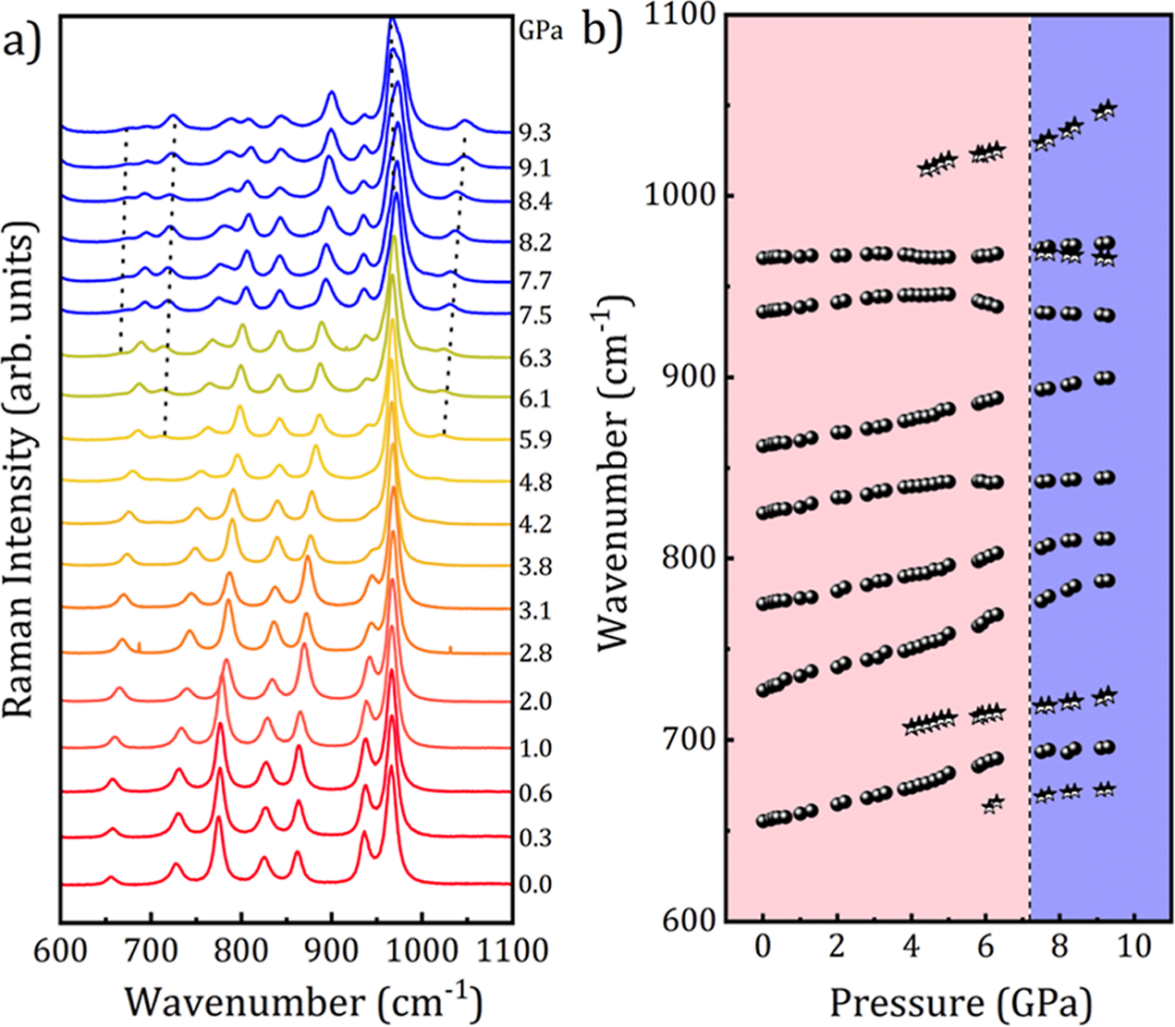
Pressure-dependent Raman spectra of Li_2_W_2_O_7_ in the following spectral regions: (a) 600–1100 cm^–1^ and Wavenumber versus pressure plots of the Raman modes of Li_2_W_2_O_7_ crystals in the corresponding spectral regions are shown in (b) 600–1100 cm^–1^.

In order to correlate the stated structural and vibrational change, it is worth to note that the vibrational assignments presented in Table S3 indicate that several Raman-active modes involve pronounced Li atomic motion, cover all Raman spectrum of the Li_2_W_2_O_7_ crystals, particularly being more accentuated in modes associated with coupled bending vibrations of the WO_6_ octahedra and LiO_4_ tetrahedra. These modes, commonly described as δ­(WO_6_ + LiO_4_) or lattice-related vibrations, reveal that the lithium sublattice plays an important role in the lattice dynamics of the structure. The large Li displacement observed in these normal modes can be rationalized by considering the intermolecular interaction topology obtained from the Hirshfeld surface analysis. In this analysis, Li···O/O···Li contacts represent the dominant contribution to the Hirshfeld surface, accounting for approximately 55.7% of the total intermolecular interactions, indicating that the lithium atoms are coordinated primarily through electrostatic interactions with surrounding oxygen atoms. Because Li–O interactions are mainly ionic and weaker than the strong covalent W–O bonds within the WO_6_ octahedra, the Li atoms experience a more flexible local environment, which allows larger vibrational amplitudes in the lattice modes. This interpretation is further supported by the Bader charge analysis (Section 3.1), which reveals that lithium atoms exhibit an effective charge of +0.90 *e*, corresponding to 90% of the formal +1 value, confirming the predominantly ionic and relatively weak character of the Li–O interactions compared to the strongly covalent W–O bonds (W effective charge of +2.98 *e*, only 50% of the formal +6 value). Additionally, the Löwdin population analysis shows that W 5*d* orbitals retain approximately 3.76 electrons despite their nominally *d* ^0^ configuration, providing direct evidence of significant O 2*p* → W 5*d* covalent donation that strengthens the WO_6_ framework while leaving the Li sublattice comparatively labile. The crystal void analysis further supports this interpretation by revealing that approximately 22.15% of the unit-cell volume consists of void space distributed throughout the framework. These void regions arise from the open structural arrangement formed by the interconnected WO_6_ octahedra and LiO_4_ tetrahedra. The presence of such voids reduces steric constraints around lithium sites and provides additional free volume that facilitates atomic displacement. Consequently, lithium atoms can oscillate within relatively open cavities of the structure, which explains why many vibrational modes show strong Li participation.

This structural flexibility also helps explain the pressure-dependent behavior observed in the Raman spectra. At low pressures, certain lattice modes that involve strong Li motion may exhibit small nonlinearities or reduced pressure coefficients because the Li atoms can adjust their positions within the available void space without significantly compressing the rigid WO_6_ framework. As pressure increases, however, the progressive reduction of the void volume restricts the mobility of the lithium ions and strengthens the Li–O interactions, leading to an overall stiffening of these vibrational modes. The calculated bulk modulus (*B* = 58.3 GPa, Section 3.2) and the moderate elastic anisotropy (A^U^ = 1.12) are consistent with the contrast between strong covalent W–O bonds and weaker ionic Li–O interactions, which underpins the pressure evolution of the Raman spectra. Overall, the combination of vibrational analysis, Hirshfeld surface topology, and void distribution indicates that the lattice dynamics of Li_2_W_2_O_7_ are strongly influenced by the lithium environment. The dominance of Li–O interactions and the presence of significant structural voids allow enhanced Li motion, which in turn contributes to the pressure-dependent vibrational response of the material. A definitive structural identification of the high-pressure phase, including its space group and refined atomic coordinates, would benefit from complementary investigations such as high-pressure X-ray diffraction or symmetry-unconstrained DFT structure searches, which we view as a natural extension of the present work.

## 4 Conclusions

In summary, the Rietveld refinement confirms a triclinic structure composed of highly distorted WO_6_ octahedra forming a connected three-dimensional framework with interstitial Li^+^ ions. DFT calculations reproduce the experimental structural parameters with good agreement. The calculated bond lengths and distortion parameters further confirm the asymmetric octahedral coordination environments characteristic of this compound. Bader charge analysis demonstrates that lithium behaves as an almost fully ionized cation, whereas tungsten exhibits a substantial covalency in its interactions with oxygen. Electronic structure calculations reveal a wide charge-transfer band gap, dominated by O 2*p* states at the valence band maximum and W 5*d* states at the conduction band minimum, in accordance with the typical electronic characteristics of *d* ^0^ tungstates. Moreover, the calculated elastic constants satisfy the mechanical stability criteria, indicating a stable lattice. The results also confirm anisotropic compressibility related to the distorted octahedral framework.

Raman spectroscopy combined with phonon calculations allows a reliable assignment of the Raman vibrational modes associated with the WO_6_ units. Under compression, most Raman modes exhibit positive pressure coefficients, reflecting progressive bond shortening and lattice stiffening. Low wavenumber modes display slight nonmonotonic behavior at low pressures, suggesting the presence of subtle conformational distortions preceding the structural transformation, associated with a more compressible Li–O sublattice embedded in an open framework.

A pressure-induced phase transition is identified between 6.3 and 7.5 GPa, as evidenced by the emergence of new Raman bands, nonlinear wavenumber shifts, and changes in pressure coefficients. This transition appears to involve symmetry reduction and rearrangement of the WO_6_ octahedral framework, rather than bond rupture, consistent with the structural adaptability of the tungstate network. At higher pressures, several modes exhibit modified pressure responses, indicating a distinct high-pressure structural regime.

Overall, this study elucidates the interaction between bonding, lattice dynamics, mechanical stability, and structural evolution under compression in Li_2_W_2_O_7_, providing valuable insights into the behavior of lithium containing tungstate frameworks under extreme conditions.

## Supplementary Material



## Notes

Scale factor optimization methodology and associated plots (Figure S1); experimental and DFT-calculated infrared spectra (Figure S2); charge density difference Δρ and Electron Localization Function maps (Figure S3); Voigt–Reuss–Hill averaging scheme for the elastic moduli; full elastic stiffness and compliance tensors in Voigt notation (Tables S1 and S2); observed and DFT-calculated vibrational wavenumbers with mode assignments (Table S3); detailed Hirshfeld surface analysis of secondary contacts (PDF)

## Notes

No unexpected, new, or significant hazards or risks are associated with the reported work.
