# Supplementary material for: Interplay between WO6 Octahedra Rigidity and Li Sub-Lattice Flexibility in Triclinic Li2W2O7: Raman, DFT, Hirshfeld Surface, and High-Pressure Studies
Source: Inorg Chem. 2026 Jun 15;65(25):14134–48. doi: 10.1021/acs.inorgchem.6c01613 (PMC13321302; doi:10.1021/acs.inorgchem.6c01613)
Supplement: Supplementary file 1 [file ic6c01613_si_001.pdf]

Supporting Information

Interplay Between WO_6 Octahedra Rigidity and Li Sub-Lattice Flexibility in Triclinic $Li_2W_2O_7$: Raman, DFT, Hirshfeld Surface, and High-Pressure Studies

José G. da Silva Filho[a,], Gilberto D. Saraiva[a], Paulo T. C. Freire[b], João G. de Oliveira Neto[c], Daniel L. M. Vasconcelos[a], Romulo S. Silva[b], Lucas S. A. Olivier[b], and Raí F. Jucá[b]*

[a]Faculty of Education, Sciences and Letters of the Sertão Central, State University of Ceará, 63902-098, Quixadá, CE, Brazil.

[b]Department of Physics, Federal University of Ceará, 60021-970, Fortaleza, CE, Brazil.

[c]Center for Social Sciences, Health, and Technology, Federal University of Maranhão, Imperatriz, Maranhão 65900-410, Brazil.

***Corresponding author:** gadelha.filho@uece.br (José G. da Silva Filho)**

Item	Description	Page
	Scale factor optimization methodology	S3
Figure S1	Scale factor optimization: RMSD vs λ, residuals, and correlation plots	S4
Figure S2	Experimental and DFT-calculated infrared (IR) spectra	S5
Figure S3	Charge density difference Δρ and ELF maps	S6
	Voigt–Reuss–Hill averaging scheme for elastic moduli	S7
Table S1	Full elastic stiffness tensor C_{ij} (GPa) in Voigt notation	S8
Table S2	Elastic compliance tensor S_{ij} (GPa^{-1}) in Voigt notation	S8
Table S3	Observed and DFT-calculated vibrational wavenumbers	S10-S11
	Detailed Hirshfeld surface analysis of secondary contacts	S12

Scale Factor Optimization Methodology

The theoretical harmonic wavenumbers obtained from DFPT calculations were scaled to account for systematic errors arising from the harmonic approximation and limitations of the exchange-correlation functional. The optimal scale factor λ was determined by minimizing the sum of squared residuals between scaled calculated wavenumbers and their experimental counterparts, following the methodology established by Scott and Radom [S1]:

$$\Delta = \Sigma_i (\lambda\omega_i^{calc} - \omega_i^{exp})^2 \qquad (S1)$$

where ω_i^{calc} and ω_i^{exp} are the ith calculated and experimental wavenumbers (in cm^{-1}), respectively, and the sum runs over all assigned mode pairs. In crystalline systems, the number of experimentally observed Raman modes often differs from the number of theoretically predicted modes due to factors such as weak Raman cross-sections, spectral overlap, or the presence of overtones and combination bands. To address this, the assignment of calculated modes to experimental peaks was performed using the Hungarian algorithm [S2], which provides the globally optimal one-to-one matching that minimizes the total frequency deviation.

The minimization of equation (S1) with respect to λ yields the analytical solution:

$$\lambda = \Sigma_i \omega_i^{calc} \omega_i^{exp} / \Sigma_i (\omega_i^{calc})^2 \qquad (S2)$$

The quality of the scaling was assessed through the root-mean-square deviation (RMSD):

$$RMSD = [\Sigma_i (\lambda\omega_i^{calc} - \omega_i^{exp})^2 / N]^{1/2} \qquad (S3)$$

where N is the number of assigned mode pairs. For $Li_2W_2O_7$, from the 33 theoretically predicted Raman-active modes and 25 experimentally observed peaks, the Hungarian algorithm successfully assigned 24 mode pairs within a maximum allowed wavenumber deviation of 50 cm^{-1}. The optimization procedure yielded an optimal scale factor of $\lambda = 1.043$, with an associated RMSD of 7.64 cm^{-1}, as illustrated by the sharp minimum in the RMSD vs. λ curve (Figure S1a). This scale factor is consistent with the known tendency of GGA functionals to underestimate phonon wavenumbers in oxide materials [S3]. The distribution of residuals ($\omega^{exp} - \lambda\cdot\omega^{calc}$) across the spectral range (Figure S1b) shows that most assigned pairs fall within ±RMSD, with no systematic trend, confirming that the single scale factor adequately corrects the calculated wavenumbers across the entire spectrum. The correlation between experimental and unscaled calculated wavenumbers (Figure S1c) reveals a linear fit with slope 1.032 and intercept 6.46 cm^{-1}, indicating the systematic underestimation by the PBE functional. After scaling, the correlation plot (Figure S1d) shows a slope of 0.9895 and intercept of 6.460 cm^{-1}, with an improved alignment along the ideal $y = x$ line. Without scaling, the RMSD was 23.10 cm^{-1}, indicating that the application of the scale factor reduced the deviation by approximately 67%.

The nine calculated modes that remained unassigned likely correspond to modes with weak Raman cross-sections below the experimental detection limit or modes whose wavenumbers overlap with more intense neighboring peaks. The single unassigned experimental peak may originate from overtones, combination bands, or impurity phases.

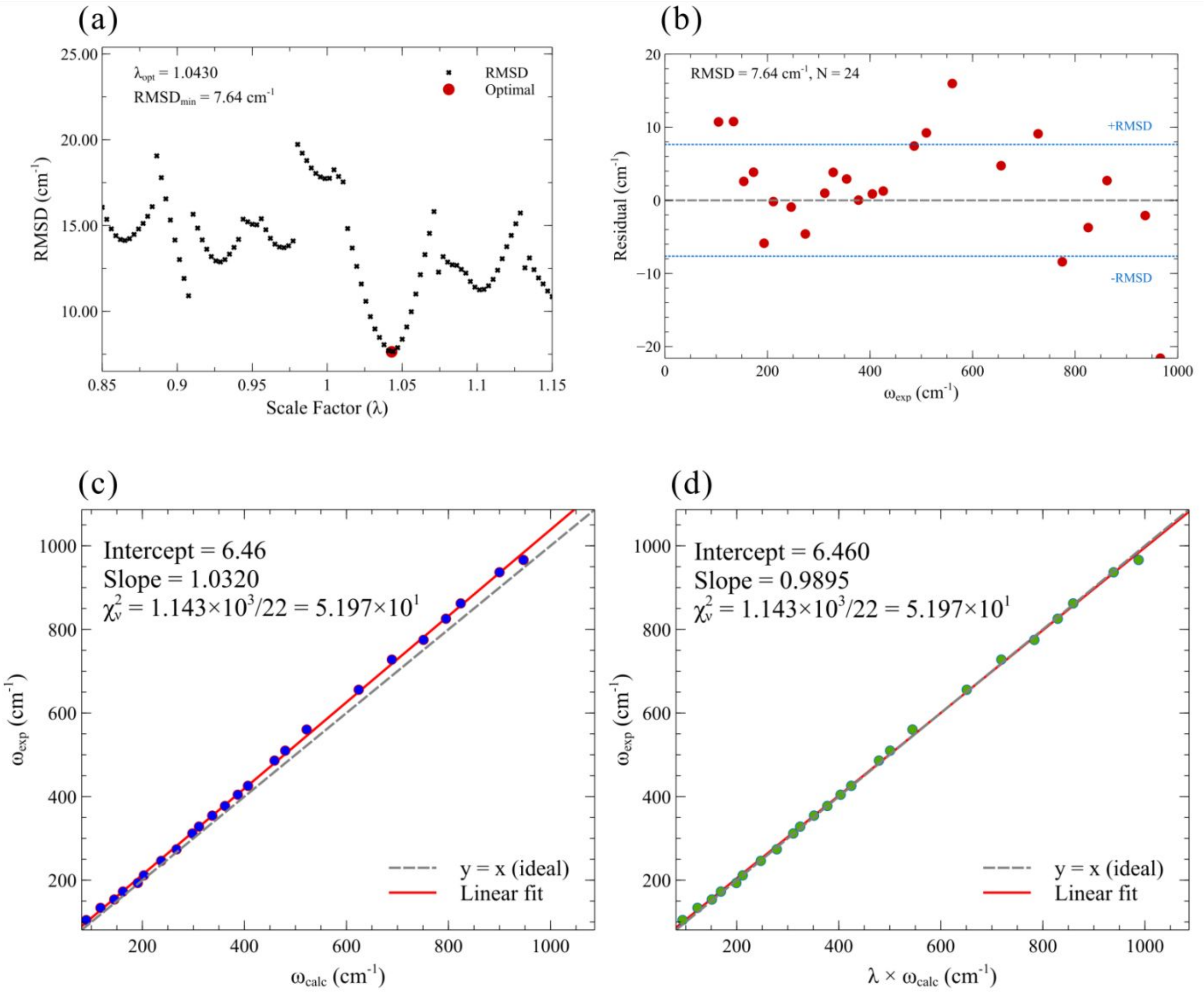

Figure S1. Scale factor optimization for $Li_2W_2O_7$ vibrational wavenumbers. (a) RMSD between scaled DFT-GGA/PBE and experimental Raman wavenumbers as a function of the scale factor λ. The optimal value λ = 1.0430 yields RMSD□$_{i}$□ = 7.64 cm^{-1}. (b) Residuals ($\omega^{exp} - \lambda\cdot\omega^{calc}$) for each assigned mode pair, plotted against the experimental wavenumber. The horizontal blue lines indicate ±RMSD. (c) Correlation between experimental and unscaled calculated wavenumbers. (d) Correlation after applying the optimal scale factor, demonstrating improved agreement with the ideal y = x relationship (N = 24 assigned pairs).

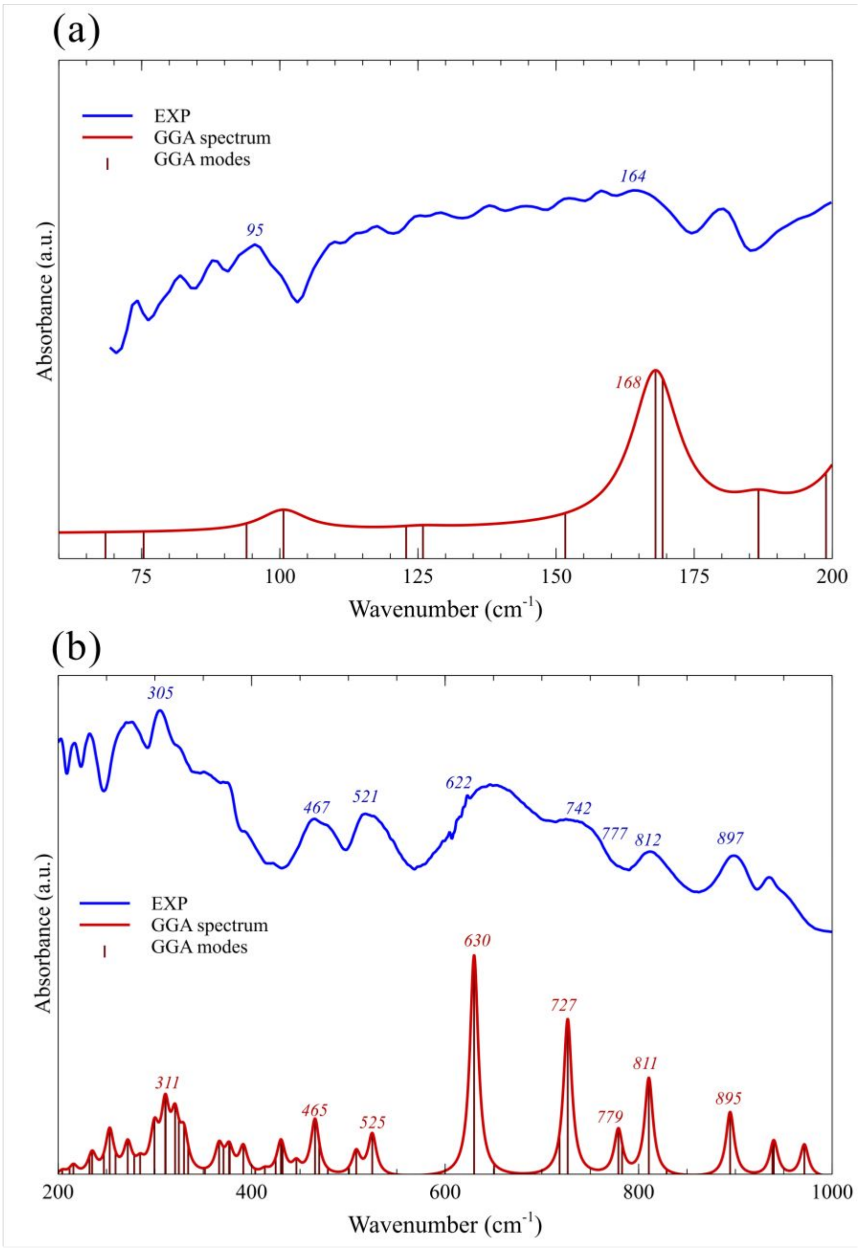

Figure S2. Comparison between the experimental (blue) and DFT-GGA/PBE calculated (red) infrared (IR) spectra of $Li_2W_2O_7$ in the spectral ranges of (a) 60–200 cm^{-1} and (b) 200–1000 cm^{-1}. Vertical bars indicate the positions and relative intensities of the calculated IR-active modes (A_u symmetry). Calculated wavenumbers were uniformly scaled by the factor $\lambda = 1.043$.

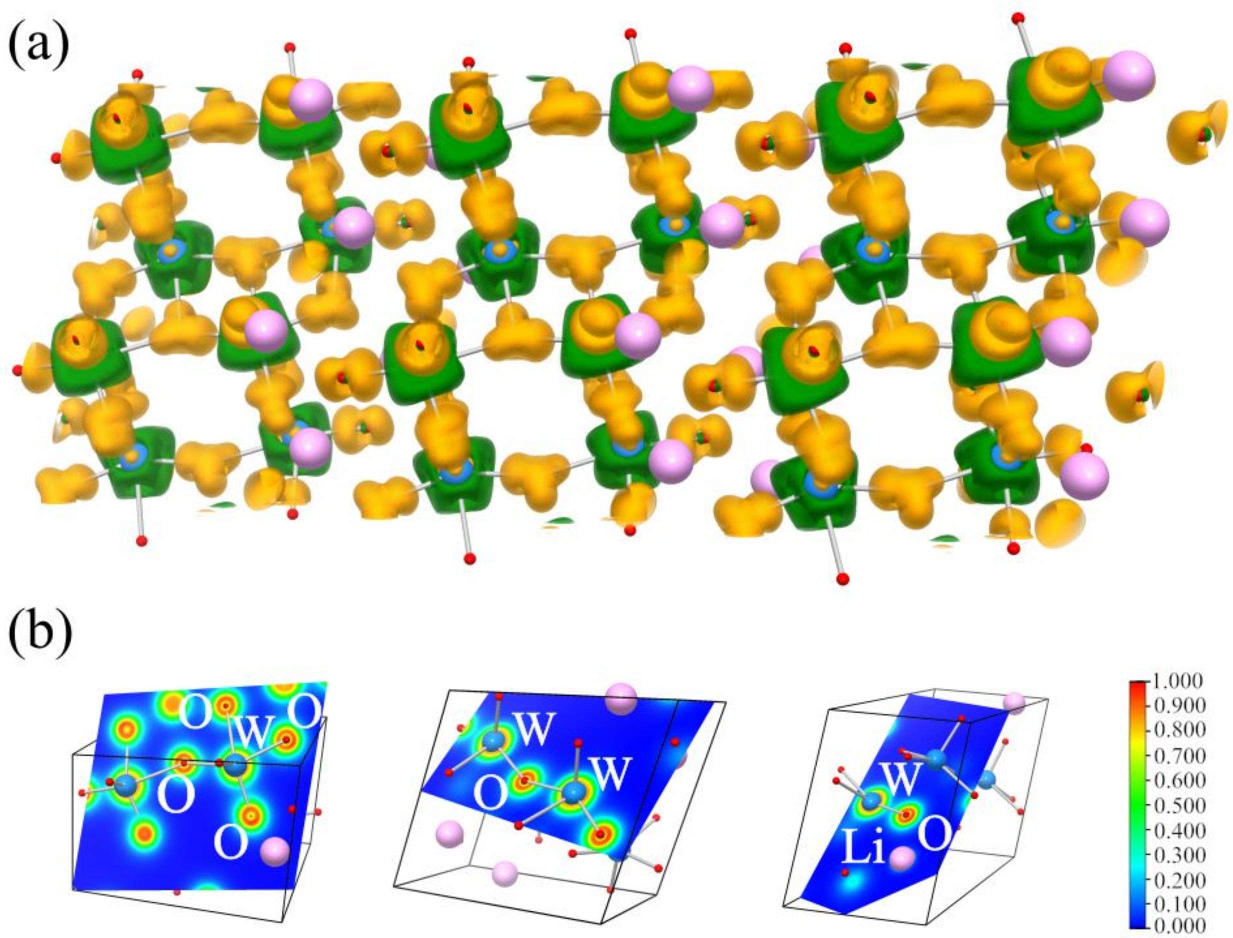

Figure S3. (a) Charge density difference Δρ for $Li_2W_2O_7$ at ±0.02 *e*/$Bohr^3$ (orange: positive; green: negative). (b) ELF maps (0–1, blue→red) on three planes: (left) WO_6 equatorial, (centre) W–O–W bridge, (right) Li–O–W. Atom colours: W (blue), Li (pink), O (red).

Voigt–Reuss–Hill Averaging Scheme for Elastic Moduli

The effective polycrystalline elastic moduli of $Li_2W_2O_7$ were derived from the single-crystal elastic stiffness tensor C_{ij} using the Voigt–Reuss–Hill (VRH) averaging scheme. The Voigt bounds assume uniform strain throughout the crystal, yielding the bulk modulus B_v and shear modulus G_v:

$$B_v = (1/9)[C_{11} + C_{22} + C_{33} + 2(C_{12} + C_{13} + C_{23})] \qquad \text{(S4)}$$

$$G_v = (1/15)[C_{11} + C_{22} + C_{33} - C_{12} - C_{13} - C_{23} + 3(C_{44} + C_{55} + C_{66})] \qquad \text{(S5)}$$

The Reuss bounds assume uniform stress and are determined from the compliance tensor $S_{ij} = C_{ij}^{-1}$:

$$B_R = 1/[(S_{11} + S_{22} + S_{33}) + 2(S_{12} + S_{13} + S_{23})] \qquad \text{(S6)}$$

$$G_R = 15/[4(S_{11} + S_{22} + S_{33}) - 4(S_{12} + S_{13} + S_{23}) + 3(S_{44} + S_{55} + S_{66})] \qquad \text{(S7)}$$

The Hill averages provide the best estimates for the polycrystalline elastic moduli:

$$B = (B_V + B_R)/2 \quad \text{and} \quad G = (G_V + G_R)/2 \qquad \text{(S8)}$$

$$E = 9BG/(3B + G) \quad \text{and} \quad \nu = (3B - 2G)/(6B + 2G) \qquad \text{(S9)}$$

The universal elastic anisotropy index A^U is defined as:

$$A^U = 5(G_V/G_R) + (B_V/B_R) - 6 \qquad \text{(S10)}$$

where $A^U = 0$ corresponds to elastic isotropy. The full elastic stiffness and compliance tensors of $Li_2W_2O_7$ are given in Tables S1 and S2, while the derived polycrystalline moduli are reported in Table 2 of the main text.

Table S1. Calculated second-order elastic stiffness constants C_{ij} (GPa) of triclinic $Li_2W_2O_7$ in Voigt notation, obtained from DFT-GGA/PBE stress–strain calculations. The triclinic structure (space group $P\underline{1}$) has 21 independent elastic constants.

	**1**	**2**	**3**	**4**	**5**	**6**
1	125.9	35.2	31.0	-0.4	7.2	-2.1
2	35.2	98.2	45.8	9.1	3.4	1.0
3	31.0	45.8	84.9	4.2	-6.0	-6.0
4	-0.4	9.1	4.2	30.1	-1.6	0.3
5	7.2	3.4	-6.0	-1.6	34.9	-11.7
6	-2.1	1.0	-6.0	0.3	-11.7	24.9

Table S2. Elastic compliance tensor S_{ij} (GPa^{-1}) of $Li_2W_2O_7$, obtained as the inverse of the stiffness tensor (Table S1).

	**1**	**2**	**3**	**4**	**5**	**6**
1	0.00929	-0.00216	-0.00250	0.00102	-0.00238	-0.00088
2	-0.00216	0.01511	-0.00778	-0.00366	-0.00405	-0.00449
3	-0.00250	-0.00778	0.01792	0.00008	0.00694	0.00766
4	0.00102	-0.00366	0.00008	0.03445	0.00203	0.00079
5	-0.00238	-0.00405	0.00694	0.00203	0.03730	0.01914
6	-0.00088	-0.00449	0.00766	0.00079	0.01914	0.05102

Table S3. Observed and DFT-calculated vibrational wavenumbers (cm^{-1}) of $Li_2W_2O_7$ with their mode assignments.

Mode	ω_{Raman} (cm^{-1})	ω_{IR} (cm^{-1})	$\omega_{calc}{}^{a}$ (cm^{-1})	Sym	Assignment$b$
1	—	—	69	A_g	$L_{bc}(WO_6) + T_{bc}(Li)$
2	—	—	75	A_g	$T_a[(WO_6) + T(Li)]$
3	105	—	94	A_g	$T[(WO_6) + T_{ac}(Li)]$, $T_a(W)$ and $T_y(O)$
4	—	90	101	A_u	$T_{bc}(WO_6) + T$ (Li)
5	134	—	123	A_g	$T[(WO_6) + (Li)]$
6	—	125	126	A_u	$T[(WO_6) + (Li)]$
7	154	—	151	A_g	$L_{bc}(WO_6) + T(Li)$
8	—	164	168	A_u	$L(WO_6) + T(Li)$
9	173	—	169	A_g	L_{bc} (WO_6) + $\delta(LiO_4)$, moderate motion of Li atoms
10	—	—	187	A_g	L_{bc} (WO_6) + $\delta(LiO_4)$, moderate motion of Li atoms
11	—	180	187	A_u	L_{bc} (WO_6) + $\delta(LiO_4)$, moderate motion of Li atoms
12	193	—	199	A_g	L_{ab} (WO_6) + $\delta(LiO_4)$, moderate motion of Li atoms
13	—	201	204	A_u	L_{bc} (WO_6) + $\delta(LiO_4)$, Strong motion of Li atoms
14	212	—	212	A_g	L_{bc} (WO_6) + $\delta(LiO_4)$, moderate motion of Li atoms
15	—	217	216	A_u	L_{bc} (WO_6) + δ $(WO_6 + LiO_4)$, Moderate motion of Li atoms
16	—	—	233	A_g	L (WO_6) + δ $(WO_6 + LiO_4)$, Moderate motion of Li atoms
17	—	229	235	A_u	L (WO_6) + δ $(WO_6 + LiO_4)$, Strong motion of Li atoms
18	246	—	247	A_g	L_{bc} (WO_6) + $\delta(LiO_4)$, Strong motion of Li atoms
19	—	239	253	A_u	δ $(WO_6 + LiO_4)$, Strong motion of Li atoms
20	—	—	260	A_g	L (WO_6) + δ $(WO_6 + LiO_4)$, Strong motion of Li atoms
21	—	274	272	A_u	δ $(WO_6 + LiO_4)$, Strong motion of Li atoms
22	274	—	278	A_g	L (WO_6) + δ $(WO_6 + LiO_4)$, Strong motion of O in the W-O-W bond plus a strong motion of Li atoms
23	—	—	285	A_u	δ $(WO_6 + LiO_4)$, Moderate motion of O in the W-O-W bond plus a strong motion of Li atoms. The system is governed by a balance of the polyhedral
24	—	—	299	A_u	δ $(WO_6 + LiO_4)$, Strong motion of Li atoms
25	—	305	311	A_u	δ $(WO_6 + LiO_4)$, Strong motion of O in the W-O-W bond plus a strong motion of Li atoms
26	312	—	311	A_g	δ $(WO_6 + LiO_4)$, Strong motion of Li atoms
27	—	—	321	A_u	δ $(WO_6 + LiO_4)$, Strong motion of Li atoms
28	328	—	324	A_g	δ $(WO_6 + LiO_4)$, Strong motion of Li atoms
29	—	326	330	A_u	δ $(WO_6 + LiO_4)$, Strong motion of Li atoms
30	—	—	335	A_g	δ $(WO_6 + LiO_4)$, Strong motion of Li atoms
31	354	—	351	A_g	δ $(WO_6 + LiO_4)$, Strong motion of Li atoms
32	—	—	366	A_u	δ $(WO_6 + LiO_4)$, Strong motion of Li atoms
33	—	—	371	A_g	δ $(WO_6 + LiO_4)$, Moderate motion of O in

					the W-O-W bond plus a strong motion of Li atoms
34	—	373	377	A_u	δ (WO_6 + LiO_4), Strong motion of O in the W-O-W bond plus a strong motion of Li atoms
35	378	—	378	A_g	δ (WO_6 + LiO_4), Strong motion of Li atoms
36	—	—	391	A_u	δ (WO_6 + LiO_4), Moderate motion of O in the W-O-W bond plus a strong motion of Li atoms.
37	405	—	404	A_g	δ (WO_6 + LiO_4), Moderate motion of O in the W-O-W bond plus a strong motion of Li atoms
38	—	400	414	A_u	δ (WO_6 + LiO_4), Moderate motion of O in the W-O-W bond plus a strong motion of Li atoms
39	426	—	424	A_g	δ (WO_6 + LiO_4), Strong motion of Li atoms
40	—	—	431	A_u	δ (WO_6 + LiO_4), Strong motion of Li atoms
41	—	—	432	A_g	δ (WO_6 + LiO_4), Strong motion of Li atoms
42	—	451	446	A_u	δ (WO_6 + LiO_4), Strong motion of O in the W-O-W bond plus a strong motion of Li atoms
43	—	467	465	A_u	δ (WO_6 + LiO_4), Strong motion of Li atoms
44	—	—	469	A_g	δ (WO_6 + LiO_4), Strong motion of O in the W-O-W bond plus a strong motion of Li atoms
45	486	—	479	A_g	δ (WO_6 + LiO_4), Strong motion of O in the W-O-W bond plus a strong motion of Li atoms
46	510	—	501	A_g	δ (WO_6 + LiO_4), Strong motion of Li atoms
47	—	474	508	A_u	δ (WO_6 + LiO_4), Strong motion of O in the W-O-W bond plus a strong motion of Li atoms
48	—	521	525	A_u	δ (WO_6 + LiO_4), Strong motion of O in the W-O -W bond plus a moderate motion of W and Li atoms.
49	560	—	544	A_g	δ (WO_6 + LiO_4), Strong motion of O in the W=O -Li bond plus a moderate motion of Li atoms.
50	—	622	630	A_u	ν_{as} (WO_6), Strong motion of O in the W-O-W bond plus a weak motion of W and Li atoms.
51	656	—	651	A_g	ν_{as} (WO_6), Strong motion of O in the W=O and W-O -W bonds plus a moderate motion of W and Li atoms.
52	728	—	719	A_g	ν_{as} (WO_6), Strong motion of O in the W=O and W-O -W bonds plus a moderate motion of W and Li atoms.
53	—	742	727	A_u	ν_{as} (WO_6), Strong motion of O in the W-O-W bond plus a moderate motion of W and Li atoms.
54	—	777	779	A_u	ν_{as} (WO_6), Strong motion of O in the W-O-W bond plus a weak motion of W and Li atoms.
55	775	—	783	A_g	ν_{as} (WO_6), Strong motion of O in the W=O bond plus a moderate motion of W and a weak motion of Li atoms.
56	—	812	810	A_u	ν_s (WO_6), Strong motion of O in the W=O bond plus a weak motion of W and Li atoms.
57	825	—	829	A_g	ν_{as} (WO_6), Strong motion of O in the W=O

					bond plus a weak motion of W and Li atoms.
58	862	—	859	A_g	ν_{as} (WO_6), Strong motion of O in the W=O bond plus a weak motion of W and Li atoms.
59	—	897	895	A_u	ν_s (WO_6) + ν_{as} (WO_6), Strong motion of O in the W=O bond plus a weak motion of W and Li atoms.
60	937	—	939	A_g	ν_s (WO_6), Strong motion of O in the W=O bond plus a weak motion of W and Li atoms.
61	—	934	940	A_u	ν_s (WO_6), Strong motion of O in the W=O bond plus a weak motion of W and Li atoms.
62	—	955	971	A_u	ν_s (WO_6), Strong motion of O in the W=O bond plus a weak motion of W and Li atoms.
63	966	—	988	A_g	ν_s (WO_6), Strong motion of O in the W=O bond plus a moderate motion of W and Li atoms.

[a] Calculated wavenumbers scaled by $\lambda = 1.043$ (RMSD = 7.64 cm^{-1}). The three acoustic translational modes ($3A_u$) are not included. [b] L = libration, T = translation, ν = stretching, ν_s = symmetric stretching, ν_{as} = asymmetric stretching, ν_{term} = terminal stretching, δ = bending, τ = torsion, γ = out-of-plane. Subscripts *a, b, c, ab, ac, bc* indicate the crystallographic axis or plane of the vibration.

Detailed Hirshfeld Surface Analysis of Secondary Contacts

O···O contacts account for 24.6% of the Hirshfeld surface area and display moderately red and white regions in the fingerprint plot, indicating a combination of short and moderate-range oxygen–oxygen interactions. These contacts represent weak van der Waals interactions and oxygen–oxygen repulsions between adjacent oxide anions in the crystal lattice. Although individually weak, the cumulative influence of these interactions contributes to the overall packing efficiency and helps define the spatial organization of the oxygen sublattice within the pyrochlore framework. The relatively high proportion of O···O contacts reflects the oxygen-rich nature of the structure and influences the material's surface chemistry, a factor of particular relevance for catalytic and gas-sensing applications where surface oxygen species play critical roles.

Li···Li contacts contribute only a minimal 0.4% to the total surface area. This negligible contribution indicates that Li^+ cations maintain sufficient separation within the structure to prevent significant cation–cation repulsions, which would otherwise destabilize the framework. The low percentage confirms the efficient distribution of Li^+ ions throughout the available tetrahedral sites of the pyrochlore lattice, optimizing electrostatic stabilization while minimizing repulsive interactions.

References

[S1] Scott, A. P.; Radom, L. Harmonic Vibrational Frequencies: An Evaluation of Hartree−Fock, Møller−Plesset, Quadratic Configuration Interaction, Density Functional Theory, and Semiempirical Scale Factors. J. Phys. Chem. 1996, 100 (41), 16502–16513. https://doi.org/10.1021/jp960976r

[S2] Kuhn, H. W. The Hungarian Method for the Assignment Problem. Nav. Res. Logist. Q. 1955, 2 (1–2), 83–97. https://doi.org/10.1002/nav.3800020109

[S3] Refson, K.; Tulip, P. R.; Clark, S. J. Variational Density-Functional Perturbation Theory for Dielectrics and Lattice Dynamics. Phys. Rev. B 2006, 73 (15), 155114. https://doi.org/10.1103/PhysRevB.73.155114
